# Rehabilitation–Cognition Integrated Care Program for Elderly With Lower Limb Fractures and Cognitive Impairment: Development and Efficacy

**DOI:** 10.1002/brb3.71184

**Published:** 2026-01-15

**Authors:** Qinfen Chen, Xiaozhen Ding, Yongmin Wei, Jiahao Wang, Mingping Zhou, Yuanyuan Chen, Yanlin Chen

**Affiliations:** ^1^ Department of Traumatology and Orthopedics The First Affiliated Hospital of Lishui University Lishui Zhejiang China; ^2^ Department of Rehabilitation The First Affiliated Hospital of Lishui University Lishui Zhejiang China; ^3^ Department of Nursing The First Affiliated Hospital of Lishui University Lishui Zhejiang China

**Keywords:** cognitive function, elderly patients, functional recovery, lower limb fractures, mild‐to‐moderate cognitive impairment, rehabilitation–cognition integrated care

## Abstract

**Objective:**

To develop and assess the efficacy of a rehabilitation–cognition integrated care (RCIC) program for elderly patients with lower limb fractures and mild‐to‐moderate cognitive impairment.

**Methods:**

A total of 128 eligible patients during January 2023 to December 2024 were randomly allocated to conventional (*n* = 64) or integrated care group (*n* = 64). Both groups received 12 weeks of intervention. Outcomes, including Fugl‐Meyer Assessment (FMA), Berg Balance Scale (BBS), Montreal Cognitive Assessment (MoCA), Functional Independence Measure (FIM), and Hospital Anxiety and Depression Scale (HADS) scores, were compared. Serum neurotrophic and neuroinflammatory markers were analyzed pre‐ and post‐intervention. Complications, fall recurrence rates, and nursing satisfaction were recorded.

**Results:**

Post‐intervention, both groups showed improved FMA, BBS, and FIM scores, with significantly greater improvement in the integrated care group (*p* < 0.05). HADS‐Anxiety (HADS‐A) and HADS‐Depression (HADS‐D) scores decreased significantly more in the integrated care group (*p* < 0.05). The integrated care group demonstrated higher MoCA scores versus both its own baseline and the conventional care group post‐intervention (*p* < 0.05). Serum BDNF and GDNF levels increased significantly in the integrated care group compared to both time‐matched controls and its baseline (*p* < 0.05), while S100‐β and IL‐6 levels decreased significantly (*p* < 0.05). The integrated care group had lower overall complication rates (*p* < 0.05), comparable fall recurrence (*p* > 0.05), and higher nursing satisfaction (*p* < 0.05).

**Conclusion:**

The RCIC program significantly enhances motor function, balance, cognition, and psychological status while reducing complications and improving satisfaction in elderly fracture patients with cognitive impairment.

## Graphical abstract

Integrated rehab–cognition program for older adults after lower limb fracture; dual‐task model combines balance/gait/strength with orientation, memory, and attention; 12‐week clinical + home delivery; improved BBS, FIM, and MoCA; reduced IL‐6 and S100‐β; feasible and acceptable; warrants multicenter and 6‐ to 12‐month follow‐up.

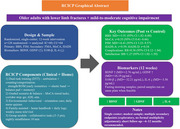

## 1 Introduction

The accelerating aging of the global population has led to a significant increase in the incidence of lower limb fractures among older adults, a trend particularly pronounced against the backdrop of prevalent osteoporosis (Feng et al. 2024; Nozaki et al. 2023). It is estimated that the occurrence of fractures involving the hip, femur, and tibia within this demographic continues to rise annually, emerging as a critical public health concern that severely compromises quality of life and life expectancy (Dong et al. 2023; Y. Wang et al. 2025). Beyond causing direct motor dysfunction and limitations in activities of daily living (ADL), lower limb fractures substantially elevate the risk of complications, including falls, refracture, deep vein thrombosis (DVT), and pulmonary infections. Consequently, these fractures contribute to high rates of disability and hospital readmissions (Tazreean et al. 2022). Furthermore, prolonged bed rest following fracture, coupled with factors such as surgical anesthesia, exerts detrimental effects on the cognitive function of older individuals (Bai et al. 2024; L. W. Wang et al. 2019).

Notably, substantial evidence indicates that the prevalence of cognitive decline is significantly higher among elderly fracture patients compared to the general aging population (Seitz et al. 2011; AbuAlrob et al. 2025; Kasai et al. 2017). A subset of patients presents with preexisting mild cognitive impairment (MCI) before surgery; postoperatively, cognitive deterioration is often exacerbated by multiple stressors, including surgical trauma, hospitalization, environmental disruption, social isolation, and pain (Bai et al. 2024). Although MCI does not meet the diagnostic criteria for dementia, it manifests as mild deficits across multiple cognitive domains (e.g., memory, executive function, language) and represents a high‐risk prodromal stage for dementia. Studies report that individuals with MCI face a three‐ to fivefold greater annual risk of progressing to dementia than cognitively intact older adults. Without intervention during fracture recovery, this cognitive vulnerability severely constrains functional rehabilitation and quality of life (Petersen et al. 2018).

Traditional nursing models primarily emphasize physical functional rehabilitation, often overlooking the dynamic nature of cognitive function and thus failing to address the comprehensive recovery needs of elderly fracture patients. In recent years, the integrated “mind–body” approach to care has gained recognition and application, underscoring the importance of simultaneous cognitive and physical functional interventions to enhance overall rehabilitation outcomes (Aftab et al. 2020; Camara et al. 2023). Dual‐task training (DTT), an intervention strategy integrating cognitive and motor tasks, has demonstrated efficacy in the rehabilitation of conditions such as Parkinson's disease, stroke, and early‐stage dementia (Garcia‐Lopez et al. 2023; Zhang et al. 2022; Xiao et al. 2023). However, research on its application in older fracture patients with comorbid MCI remains relatively scarce, and systematic individualized intervention protocols are lacking.

Against this backdrop, this study targets older adults with lower limb fractures and comorbid mild‐to‐moderate cognitive impairment. We aim to construct a multifaceted rehabilitation–cognition integrated care (RCIC) program incorporating cognitive–motor DTT, real‐world scenario simulation, and family‐assisted management. This program will be evaluated for its effects on improving motor function, cognitive status, emotional responses, and functional independence. Furthermore, we will explore the underlying mechanisms through the biological validation of neurotrophic factors and neuroinflammatory markers. The goal is to provide this vulnerable population with a more scientific, comprehensive, and personalized care pathway, thereby delaying cognitive decline progression, facilitating functional recovery, and offering theoretical and technical support for clinical practice.

## 2 Materials and Methods

### 2.1 Baseline Characteristics

A total of 128 elderly patients with lower limb fractures and comorbid mild‐to‐moderate cognitive impairment, admitted to our hospital between January 2023 and December 2024, were randomly assigned using a random number table method to either the conventional care group (*n* = 64) or the integrated care group (*n* = 64). The baseline characteristics of both groups were comparable, with no statistically significant differences observed in any of the parameters detailed in Table 1 (all *p* > 0.05).

**TABLE 1:** Comparison of baseline characteristics between groups.

Parameter	Integrated care group (*n* = 64)	Conventional care group (*n* = 64)	*Z/t/χ* ^2^	*p*
Age (years)	74.00 (71.00, 77.00)	75.50 (71.25, 79.00)	−1.709	0.087
BMI (kg/m^2^)	22.06 ± 2.55	21.85 ± 2.48	0.466	0.642
Time from fracture to admission (days)	2.00 (2.00, 3.00)	3.00 (2.00, 3.00)	−0.663	0.507
Bone mineral density T‐score	−2.60 (−2.88, −2.23)	−2.50 (−2.88, −2.20)	−0.519	0.604
Female (*n* [%])	39 (60.94%)	36 (56.25%)	0.290	0.590
Educational level (*n* [%])	—	—	0.862	0.835
Illiterate	13 (20.31%)	15 (23.44%)	—	—
Primary school	27 (42.19%)	24 (37.50%)	—	—
Secondary school	16 (25.00%)	19 (29.69%)	—	—
High school or above	8 (12.50%)	6 (9.37%)	—	—
Fracture type (*n* [%])	—	—	0.533	0.465
Hip fracture	42 (65.63%)	38 (59.38%)	—	—
Femoral/tibial fracture	22 (34.37%)	26 (40.62%)	—	—
Hypertension (*n* [%])	39 (60.94%)	34 (53.13%)	0.797	0.372
Diabetes (*n* [%])	6 (9.37%)	7 (10.94%)	0.086	0.770
Osteoporosis (*n* [%])	43 (67.19%)	45 (70.31%)	0.145	0.703

Abbreviation: BMI, body mass index (kg/m^2^).

### 2.2 Inclusion Criteria

Patients were included if they met the following criteria: (1) aged between 60 and 80 years, consistent with the World Health Organization (WHO) definition of older adults (Beard et al. 2016); (2) radiologically confirmed diagnosis of a lower limb fracture (involving the hip, femur, or tibia); (3) comorbid mild‐to‐moderate cognitive impairment, defined as a Montreal Cognitive Assessment (MoCA) score ranging from 16 to 25 points, meeting the international consensus criteria for cognitive frailty (Nasreddine et al. 2005; Kelaiditi et al. 2013); (4) vital sign stability permitting tolerance of rehabilitation training; (5) possession of basic ADL independence prior to the fracture; (6) absence of severe language or hearing impairments allowing cooperation with cognitive assessments and nursing interventions; and (7) provision of written informed consent by the patient following approval of the study protocol by the Ethics Committee of our hospital.

### 2.3 Exclusion Criteria

Patients were excluded from the study if they met any of the following criteria: (1) presence of concurrent fractures at other sites or open fractures; (2) severe cognitive impairment or dementia, defined as a score < 10 points or a clinical diagnosis of Alzheimer's disease or other dementia; (3) severe dysfunction of major organ systems (e.g., class IV heart failure, end‐stage renal disease); (4) comorbid major psychiatric disorders (e.g., schizophrenia, major depressive disorder); (5) history of major surgery, chemotherapy, or radiotherapy within the preceding 3 months; (6) current participation in another interventional rehabilitation research; or (7) an estimated life expectancy of less than 6 months or inability to complete the follow‐up protocol.

### 2.4 Withdrawal and Dropout Criteria

Patients were withdrawn from the study or classified as dropouts if they met any of the following criteria: (1) missing essential research data; (2) experiencing a serious adverse event during the intervention period requiring urgent medical intervention (e.g., acute myocardial infarction, stroke, septic shock); (3) experiencing clinical deterioration (e.g., fracture nonunion, severe infection) precluding continuation in the care protocol; or (4) voluntary withdrawal requested by the patient or family, or loss to follow‐up. Within the integrated care group, 64 patients were initially enrolled. During the intervention period, 4 were lost to follow‐up, resulting in 60 patients ultimately completing the study (completion rate: 93.75%). In the conventional care group, all 64 enrolled patients completed the study without any withdrawals or dropouts (completion rate: 100.00%). The retention rate exceeded 90% in both groups, meeting the data integrity requirements for clinical trials.

### 2.5 Nursing Interventions

(1) Conventional care group: Patients received standard nursing care, encompassing: (i) Basic nursing care, including (a) twice‐daily monitoring of vital signs (temperature, blood pressure, heart rate); (b) pain management involving prescribed non‐steroidal anti‐inflammatory drugs (e.g., ibuprofen) supplemented by ice application and positional adjustments; and (c) complication prevention strategies such as repositioning every 2 h to prevent pressure ulcers and intermittent pneumatic compression therapy for DVT prophylaxis. (ii) Basic rehabilitation training, initiated with (a) bedside passive range of motion (ROM) exercises for the hip, knee, and ankle joints (commenced on postoperative Day 3, administered twice daily for 15 min per session); (b) isometric muscle strengthening exercises targeting the quadriceps and gluteal muscles (initiated at 1 week postoperatively, performed in 3 sets of 10 repetitions daily); and (c) progressive gait training with a walker, advancing from bedside standing to assisted ambulation (initiated at 2 weeks postoperatively, conducted once daily for 10 min). (iii) Health education, covering (a) dietary guidance emphasizing high‐calcium and high‐protein intake; (b) medication adherence support for calcium and vitamin D supplementation, adhering strictly to the prescribed dosage and schedule; and (c) pre‐discharge fall prevention education for patients and caregivers focusing on home safety modifications.

(2) Integrated care group: In addition to the conventional care protocol, patients received an individualized RCIC intervention, tailored to clinical feasibility and comprising the following components: (i) DTT: (a) Ambulation enhancement: Patients performed cognitive tasks (e.g., object counting, simple item categorization) while ambulating with a walker, with progressive increases in distance and speed. Obstacle negotiation tasks (e.g., stepping over soft mats, navigating around cones) were introduced starting at Week 4 postoperatively (twice daily, 15 min/session) to improve lower limb coordination and dynamic balance. (b) Strength and ROM training: Early phase (Weeks 1–2 postoperatively): Primarily isometric contractions synchronized with number repetition tasks (e.g., “count 1–5 during contraction, 5–1 during relaxation”; 3 sets of 10 repetitions daily). Intermediate phase (Weeks 3–6): Added resistance training (elastic band‐assisted hip abduction, knee extension) performed concurrently with multistep command execution (e.g., “lift leg→hold for 3 s→lower”; 2 sets of 8–12 repetitions daily). Late phase (Weeks 7–12): Integrated single‐leg stance on a balance pad (with handrail support) combined with short‐term memory tasks (e.g., recalling three object names; held for 10–20 s, repeated 4–6 times daily). (ii) Graded reality‐based scenario simulation and balance training: (a) Lower‐level tasks (MoCA 16–19): (1) static seated balance combined with simple upper‐limb cognitive activities (e.g., puzzle assembly) that require trunk stability; (2) dynamic corridor balance using a walk–pause–answer paradigm—patients pause every five steps to answer brief orientation questions—under nurse supervision. (b) Higher‐level tasks (MoCA 20–25): (1) complex balance within simulated ADL settings in the rehabilitation room (e.g., walking while carrying a cup, ascending/descending low steps) performed concurrently with calculation tasks (e.g., serial subtraction of 3 from 20; three times weekly, 20 minutes per session); (2) functional relearning via route‐planning with hospital maps, requiring ambulation from the ward to a designated location (e.g., nursing station) and verbal description of route details to train spatial navigation and gait control. (iii) Environmental–behavioral adaptive interventions: (a) Enhanced orientation cues via large‐print calendars, clocks, and directional signs in wards, supplemented by daily morning time and space orientation quizzes conducted by nurses (e.g., “What floor are you on?,” “What is your next scheduled activity?”). (b) Reality orientation and ADL retraining within simulated home environments (e.g., dressing, grooming), incorporating error correction tasks (e.g., identifying and rectifying deliberately misplaced items; thrice weekly, 20 min/session). (iv) Family‐assisted rehabilitation–cognition management: (a) Provision of a home‐based Cognitive–Motor Task Handbook (Appendix S1) outlining daily orientation quizzes, memory games, and safe walking challenges. Family members documented task completion daily. (b) Weekly nurse‐led home visits (or video consultations) to adjust task difficulty based on patient progress (e.g., increasing step complexity, reducing verbal cues). (v) Group rehabilitation–cognition interaction module: (a) Collaborative group tasks (e.g., puzzle relay, group command‐following exercises) for 3–5 patients to foster social interaction and executive function training (twice weekly, 30 min/session). (b) Brief mindfulness‐based intervention integrating guided breathing with positive imagery (e.g., “visualize walking in a park”) for anxiety and depression alleviation (conducted for 10 min each evening). Details of the RCICP structure can be found in Table 2. Outcome measures for both groups were assessed following 12 weeks of continuous intervention.

**TABLE 2:** Summary of the RCICP components.

Component	Subcomponent/examples	Dosage/frequency	Timeline	Setting	MoCA tier
DTT	Ambulation enhancement (walker + cognitive tasks: counting, categorization; add obstacles from Week 4)	2×/day, 15 min/session; progressive distance/speed; obstacles from Week 4	Weeks 1–12	Clinical (ward/rehab corridor)	All (MoCA 16–25)
DTT	Strength and ROM—Early: isometric with number repetition (count 1–5)	3 sets × 10 reps daily	Weeks 1–2	Clinical/home	All (MoCA 16–25)
DTT	Strength and ROM—Intermediate: elastic band hip abduction, knee extension + multistep commands	2 sets × 8–12 reps daily	Weeks 3–6	Clinical/home	All (MoCA 16–25)
DTT	Strength and ROM—Late: single‐leg stance on balance pad (with handrail) + short‐term memory (recall 3 items)	Hold 10–20 s, repeat 4–6×/day	Weeks 7–12	Clinical/home	All (MoCA 16–25)
Graded scenario simulation and balance	Lower level (MoCA 16–19): seated static balance + cognitive (puzzles); corridor stop–go with orientation Q&A	Supervised; short bouts → ∼20 min	Weeks 1–12	Clinical (ward/corridor)	MoCA 16–19
Graded scenario simulation and balance	Higher level (MoCA 20–25): ADL‐simulated complex balance + calculation; route planning using hospital maps	3×/week, 20 min/session; plus route‐walking	Weeks 1–12	Rehab room/clinical spaces	MoCA 20–25
Environmental–behavioral adaptations	Orientation cues (calendars, clocks, signs) + daily morning orientation quizzes by nurses	Daily	Weeks 1–12	Ward	All (MoCA 16–25)
Environmental–behavioral adaptations	Reality orientation and ADL retraining; error correction tasks (identify/rectify misplaced items)	3×/week, 20 min/session	Weeks 1–12	Rehab room	All (MoCA 16–25)
Family‐assisted rehab–cognition	Home‐based Cognitive–Motor Task Handbook (Appendix S1); daily orientation/memory; safe walking; family logs	Daily per handbook	Weeks 1–12	Home	All (MoCA 16–25)
Family‐assisted rehab–cognition	Weekly nurse home visit/video to titrate difficulty (increase step complexity, reduce cues)	1×/week, 20–30 min	Weeks 1–12	Home/telehealth	All (MoCA 16–25)
Group rehab–cognition module	Collaborative group tasks (puzzle relay, group command‐following) for 3–5 patients	2×/week, 30 min/session	Weeks 1–12	Rehab room	All (MoCA 16–25)
Group rehab–cognition module	Mindfulness: guided breathing + positive imagery (e.g., visualize walking in a park)	10 min each evening	Weeks 1–12	Ward/home	All (MoCA 16–25)

### 2.6 Outcome Measures

(1) Motor function: Motor recovery was assessed using the lower extremity subscale of the Fugl‐Meyer Assessment (FMA) (Fugl‐Meyer et al. 1975), which evaluates joint ROM, muscle strength, and coordination. Scores range from 0 to 34, with higher scores indicating superior motor function. Assessments were performed before the intervention and after 12 weeks of nursing care.

(2) Balance capacity: Static and dynamic balance were quantified using the Berg Balance Scale (BBS) (Berg et al. 1992). This scale comprises 14 tasks (e.g., standing, turning), with total scores ranging from 0 to 56. Higher scores denote better balance performance. Assessments occurred at baseline and post‐intervention (12 weeks).

(3) Cognitive function: Global cognitive function across seven domains (including visuospatial abilities, memory, and language) was evaluated using the MoCA (Nasreddine et al. 2005). Total scores range from 0 to 30, with the established baseline range for mild‐to‐moderate cognitive impairment in this study being 16–25 points. MoCA assessments were conducted at baseline and 12 weeks.

(4) Functional independence: The level of independence in ADL and social functioning was analyzed using the Functional Independence Measure (FIM) (Keith et al. 1987). The FIM encompasses both motor (e.g., walking, toileting) and cognitive (e.g., problem‐solving) subscales, with total scores ranging from 18 (lowest independence) to 126 (complete independence). Higher scores reflect greater functional independence. Measurements were taken pre‐ and post‐intervention (12 weeks).

(5) Psychological status: Emotional states, specifically anxiety and depression, were assessed using the respective subscales of the Hospital Anxiety and Depression Scale (HADS) (Zigmond and Snaith 1983): the Anxiety subscale (HADS‐A) and the Depression subscale (HADS‐D). Each subscale consists of 7 items, with scores ranging from 0 to 21 for each. Higher scores indicate more severe symptoms of anxiety or depression. HADS evaluations were performed at baseline and after 12 weeks of care.

(6) Neurotrophic factors: Serum levels of brain‐derived neurotrophic factor (BDNF) and glial cell line‐derived neurotrophic factor (GDNF) were measured in both groups before the intervention and after 12 weeks of care. Blood sampling was standardized: all specimens were collected in the morning (approximately 07:00–09:00) after an overnight fast (≥ 8 h); participants rested in a seated position for ∼10 min before venipuncture, avoided vigorous exercise and alcohol for 24 h, and morning medications were recorded (essential drugs not withheld). After allowing the blood to clot at room temperature for 30 min, serum was separated by centrifugation at 3000 rpm for 15 min within 60 min of collection. The separated serum was aliquoted into microcentrifuge (EP) tubes and stored at −80°C until analysis. Serum factor concentrations were determined using enzyme‐linked immunosorbent assay (ELISA) kits purchased from Shanghai EK‐Bioscience Biotechnology Co. Ltd.

(7) Neuroinflammatory markers: Serum levels of central nervous system‐specific protein S100‐β (S100‐β) and interleukin‐6 (IL‐6) were quantified in both groups at baseline and post‐intervention (12 weeks). The same standardized phlebotomy conditions were applied (morning collection after overnight fasting, pre‐draw seated rest, pre‐analytic restrictions, and timed processing within 60 min) (Section 2.6 [6]), fasting peripheral venous blood samples were processed to obtain serum, which was aliquoted, stored at −80°C, and subsequently analyzed for S100‐β and IL‐6 concentrations via ELISA using kits from the same supplier (Shanghai EK‐Bioscience Biotechnology Co. Ltd.).

(8) Safety and nursing quality: The following safety and quality metrics were recorded: (i) Overall incidence of complications during the care period, including delirium, pressure ulcers, pulmonary infection, urinary tract infection, thrombosis, delayed fracture union or nonunion, and joint stiffness and muscle atrophy; (ii) Fall recurrence rate; and (iii) Nursing satisfaction, assessed using a 5‐point Likert scale ranging from 1 (very dissatisfied) to 5 (very satisfied). Satisfaction rates were calculated by determining the proportion of patients reporting “Satisfied” (scores 4–5), “Neutral” (score 3), and “Dissatisfied” (scores 1–2) within each group.

### 2.7 Statistical Analysis

Statistical analyses were performed using SPSS software (version 27.0). Categorical data were presented as (*n* [%]) and compared using the chi‐square (*χ* ^2^) test. Quantitative data demonstrating a normal distribution were expressed as mean ± standard deviation ($\bar{x}$ ± *s*) and analyzed using either the independent samples or the paired samples *t*‐test. Quantitative data not conforming to a normal distribution were presented as M (p25, p75) and analyzed using the Mann–Whitney *U* test or the Wilcoxon signed‐rank test. For normally distributed continuous outcomes, the between‐group mean difference (MD) with 95% CI; for non‐normal continuous outcomes, the rank‐biserial correlation (r) with 95% CI; and for categorical outcomes, the risk ratio (RR) with 95% CI. A *p* value of less than 0.05 was considered statistically significant for all analyses. BBS and FIM were prespecified as primary outcomes, and all other measures were treated as secondary/exploratory. Given the exploratory nature across multiple secondary endpoints, no formal multiplicity correction was applied; accordingly, effect sizes with 95% CIs are emphasized, and *p* values are interpreted cautiously.

## 3 Results

### 3.1 Comparison of Motor Function and Balance Capacity

Prior to intervention, no significant differences were observed between the integrated care and conventional care groups in FMA or BBS scores (*p* > 0.05). Following the intervention period, both groups exhibited significant improvements in FMA and BBS scores compared to their respective baselines. Notably, the integrated care group demonstrated significantly greater improvements in both outcome measures relative to the conventional care group (FMA: r = 0.39, 95% CI 0.27–0.51; BBS: MD = 3.91 points, 95% CI 1.82–6.00) (*p* < 0.05) (Table 3).

**TABLE 3:** Comparison of motor function and balance capacity between groups (scores).

Time	Integrated care group (*n* = 60)	Conventional care group (*n* = 64)	*Z/t*	*p*
Pre‐intervention				
FMA	10.00 (8.00, 12.00)	10.00 (8.00, 13.00)	0.782	0.434
BBS	22.25 ± 5.18	21.89 ± 4.92	0.396	0.693
Post‐intervention				
FMA	25.00 (22.00, 28.00)^a^	23.00 (20.00, 25.00)^a^	3.736	< 0.001
BBS	41.88 ± 5.89^a^	37.97 ± 5.87^a^	3.707	< 0.001

*Note*: Compared with pre‐intervention assessment within the same group, ^a^ *p* < 0.05.

### 3.2 Comparison of Cognitive Function and Independence

At baseline, no statistically significant differences were observed in MoCA or FIM scores between the integrated care group and the conventional care group (*p* > 0.05). Following the intervention, the integrated care group demonstrated significantly higher MoCA scores compared to both its own baseline assessment (*p* < 0.05) and the post‐intervention scores of the conventional care group (r = 0.55, 95% CI 0.41–0.66) (*p* < 0.05). In contrast, the conventional care group exhibited no significant change in MoCA scores from baseline (*p* > 0.05). Regarding functional independence, both groups showed significant increases in FIM scores post‐intervention. However, the integrated care group achieved significantly higher FIM scores than the conventional care group (MD = 6.41 points, 95% CI 3.25–9.57) (*p* < 0.05) (Table 4).

**TABLE 4:** Comparison of cognitive function and independence between groups (scores).

Time	Integrated care group (*n* = 60)	Conventional care group (*n* = 64)	*Z/t*	*p*
Pre‐intervention				
MoCA	19.50 (18.00, 21.00)	20.00 (18.00, 21.00)	0.215	0.830
FIM	72.05 ± 7.63	71.94 ± 7.34	0.084	0.933
Post‐intervention				
MoCA	23.00 (21.00, 25.00)^a^	20.00 (18.00, 22.00)	6.109	< 0.001
FIM	98.27 ± 9.15^a^	91.86 ± 8.56^a^	4.028	< 0.001

*Note*: Compared with baseline assessment within the same group, ^a^ *p* < 0.05.

### 3.3 Comparison of Psychological Outcomes

At baseline, no significant differences existed between the integrated care group and the conventional care group in scores for either HADS‐A or HADS‐D (*p* > 0.05). Following the intervention period, both groups demonstrated significant reductions in HADS‐A and HADS‐D scores compared to their respective baselines. Notably, the integrated care group achieved significantly lower scores on both psychological measures than the conventional care group (HADS‐A: r = 0.59, 95% CI 0.46–0.69; HADS‐D: r = 0.54, 95% CI 0.40–0.65) (*p* < 0.05) (Table 5).

**TABLE 5:** Comparison of psychological outcomes between groups (scores).

Time	Integrated care group (*n* = 60)	Conventional care group (*n* = 64)	*Z*	*p*
Pre‐intervention				
HADS‐A	12.00 (10.00, 14.00)	12.00 (10.00, 14.00)	0.028	0.978
HADS‐D	11.50 (10.00, 13.00)	11.00 (10.00, 13.00)	0.137	0.891
Post‐intervention				
HADS‐A	6.00 (5.00, 7.00)^a^	9.00 (7.00, 10.00)^a^	6.554	< 0.001
HADS‐D	5.00 (4.00, 7.00)^a^	8.00 (6.00, 9.00)^a^	6.016	< 0.001

*Note*: Compared with baseline assessment within the same group, ^a^ *p* < 0.05.

### 3.4 Comparison of Serum Neurotrophic Factor Levels

At baseline assessment, no statistically significant differences were observed in serum levels of BDNF or GDNF between the integrated care and conventional care groups (*p* > 0.05). Following the intervention period, the integrated care group demonstrated significantly elevated serum BDNF and GDNF levels compared to both its own baseline measurements (*p* < 0.05) and the post‐intervention levels of the conventional care group (BDNF: MD = 2.76 ng/mL, 95% CI 1.49–4.03; GDNF: MD = 16.19 pg/mL, 95% CI 9.51–22.87) (*p* < 0.05). Conversely, the conventional care group exhibited no significant changes in either neurotrophic factor relative to baseline (*p* > 0.05) (Table 6).

**TABLE 6:** Comparison of serum neurotrophic factor levels between groups.

Time	Integrated care group (*n* = 60)	Conventional care group (*n* = 64)	*t*	*p*
Pre‐intervention				
BDNF (ng/mL)	18.32 ± 2.93	18.44 ± 3.74	0.390	0.698
GDNF (pg/mL)	85.50 ± 12.89	84.76 ± 15.00	0.116	0.908
Post‐intervention				
BDNF (ng/mL)	22.34 ± 3.20^a^	19.58 ± 4.01	3.804	< 0.001
GDNF (pg/mL)	106.37 ± 17.20^a^	90.18 ± 20.67	4.080	< 0.001

*Note*: Compared with baseline assessment within the same group, ^a^ *p* < 0.05.

### 3.5 Comparison of Serum Neuroinflammatory Markers

Baseline assessments revealed no statistically significant differences in serum levels of S100‐β or IL‐6 between the integrated care and conventional care groups (*p* > 0.05). Following the intervention, the integrated care group exhibited significant reductions in both serum S100‐β and IL‐6 levels relative to its own pre‐intervention measurements (*p* < 0.05), concurrently demonstrating lower post‐intervention concentrations than those observed in the conventional care group (S100‐β: MD = −32.21 pg/mL, 95% CI −43.62 to −20.80; IL‐6: MD = −3.56 pg/mL, 95% CI −4.10 to −3.02) (*p* < 0.05). In contrast, the conventional care group showed no significant alterations in either neuroinflammatory marker compared to baseline values (*p* > 0.05) (Table 7).

**TABLE 7:** Comparison of serum neuroinflammatory markers between groups (pg/mL).

Time	Integrated care group (*n* = 60)	Conventional care group (*n* = 64)	*t*	*p*
Pre‐intervention				
S100‐β	215.58 ± 32.26	214.11 ± 36.13	0.181	0.856
IL‐6	8.42 ± 1.90	8.62 ± 2.00	0.753	0.453
Post‐intervention				
S100‐β	172.63 ± 27.94^a^	204.84 ± 35.97	5.409	< 0.001
IL‐6	4.38 ± 1.10^a^	7.94 ± 1.87	12.655	< 0.001

*Note*: Compared with baseline assessment within the same group, ^a^ *p* < 0.05.

### 3.6 Comparison of Complications and Fall Recurrence Rates

The integrated care group demonstrated a significantly lower overall complication rate compared to the conventional care group (6.67% vs. 18.75%; RR = 0.36, 95% CI 0.12–1.04, *p* < 0.05). However, no statistically significant difference was observed in fall recurrence rates between the integrated and conventional care groups (1.67% vs. 4.69%; *p* > 0.05) (Table 8).

**TABLE 8:** Comparison of complications and fall recurrence rates between groups (*n* [%]).

Indicator	Integrated care group (*n* = 60)	Conventional care group (*n* = 64)	*χ* ^2^	*p*
Delirium	2 (3.33%)	4 (6.25%)	—	—
Pressure ulcers	0 (0.00%)	1 (1.56%)	—	—
Pulmonary infection	1 (1.67%)	3 (4.69%)	—	—
Urinary tract infection	1 (1.67%)	2 (3.13%)	—	—
Thrombosis	0 (0.00%)	1 (1.56%)	—	—
Delayed/nonunion of fracture	0 (0.00%)	1 (1.56%)	—	—
Joint stiffness and muscle atrophy	0 (0.00%)	0 (0.00%)	—	—
Overall complication rate	4 (6.67%)	12 (18.75%)	4.023	0.045
Fall recurrence rate	1 (1.67%)	3 (4.69%)	0.196	0.658

### 3.7 Comparison of Nursing Satisfaction

The integrated care group demonstrated significantly higher overall nursing satisfaction compared to the conventional care group (81.67% vs. 64.06%; RR = 1.27, 95% CI 1.02–1.59), with a statistically significant difference (*p* < 0.05), as seen in Table 9.

**TABLE 9:** Comparison of nursing satisfaction between groups (*n* [%]).

Satisfaction level	Integrated care group (*n* = 60)	Conventional care group (*n* = 64)	*χ* ^2^	*p*
Dissatisfied	2 (3.33%)	7 (10.94%)	1.650	0.199
Neutral	9 (15.00%)	16 (25.00%)	1.924	0.165
Satisfied	49 (81.67%)	41 (64.06%)	4.822	0.028

## 4 Discussion

The advent of an aging society has rendered the effective enhancement of holistic rehabilitation outcomes in elderly fracture patients, particularly the synergistic recovery of cognitive and motor functions, a critical priority in nursing research. A substantial proportion of geriatric patients sustaining lower limb fractures concurrently present with mild‐to‐moderate cognitive impairment. Without targeted intervention, this population frequently experiences progressive cognitive decline, protracted motor functional recovery, and diminished quality of life (Tseng et al. 2021; Wu et al. 2024). Building upon conventional nursing protocols, the present study developed and implemented a systematic RCIC program. Our findings demonstrated that this integrated approach conferred significant advantages in enhancing patients’ motor function, cognitive performance, psychological status, and functional independence, while simultaneously reducing complication rates and elevating nursing satisfaction.

Regarding motor function and balance capacity, the integrated care group exhibited markedly superior FMA and BBS scores compared to conventional care recipients. This improvement may be attributed to the implementation of DTT and real‐world scenario simulations, which effectively augmented lower limb muscle strength, dynamic balance, and coordinative abilities. Traditional rehabilitation regimens predominantly emphasize passive mobilization or isolated limb exercises, often failing to adequately engage patients’ attentional resources and executive functions (Sadeghi et al. 2021). Our intervention incorporated cognitively embedded tasks, such as counting exercises, categorization activities, and route planning, during physical training. This integration enhances neuromuscular coordination, increases treatment adherence, and promotes the transfer of acquired skills to ADL, thereby accelerating functional recovery. These observations align with existing literature supporting the effect of cognitive–motor dual‐task paradigms in optimizing rehabilitation efficiency (Ali et al. 2022; Kuo et al. 2022; Zhou et al. 2025).

In the cognitive domain, the integrated care group manifested significantly elevated MoCA scores post‐intervention relative to conventional care counterparts. This underscores the RCIC program's robust efficacy in facilitating cognitive enhancement. The progressive escalation of task difficulty, coupled with family participation and environmental enrichment strategies, including spatial orientation cues, object recall games, and ADL reconstruction training, likely contributed to functional activation within prefrontal cortical and hippocampal regions. Such neurocognitive engagement potentially underlies improvements in attention, memory consolidation, and executive functioning (Kusleikiene et al. 2025). Furthermore, the incorporation of group‐based interactions and mindfulness‐based interventions, to some extent, mitigated cognitive load while enhancing the intrinsic motivation and sustainability of cognitive training (Udvardi et al. 2024).

Psychological assessments revealed significantly lower HADS‐A and HADS‐D subscale scores in the integrated care group than in the conventional care group. Geriatric fracture patients frequently experience anxiety and depressive symptomatology, often stemming from apprehensions regarding functional prognosis, environmental unfamiliarity during hospitalization, and distress over perceived cognitive deterioration (Jaatinen et al. 2022). Our multimodal approach, encompassing mindfulness practices, structured group activities, and family involvement, effectively ameliorated negative emotional states by cultivating a supportive therapeutic milieu. This psychologically attuned framework encouraged more active rehabilitation participation, thereby indirectly improving mental health outcomes. These findings underscore the imperative of integrating psychosocial support alongside physical rehabilitation to achieve comprehensive recovery objectives.

Notably, observed alterations in neurotrophic and inflammatory biomarkers provide mechanistic insights into the intervention's physiological effects. Post‐intervention elevations in BDNF and GDNF levels, as critical factors regulating neural plasticity and cognitive function, within the integrated care group suggest enhanced neural network and synaptic remodeling (Laksmidewi et al. 2019; Gadad et al. 2021). Concurrent reductions in IL‐6 and S100‐β, as representative markers of neuroinflammatory status, indicate that the intervention effectively suppresses chronic neuroinflammatory responses post‐fracture (Leonardo and Fregni 2023), collectively supporting the neuroprotective properties of RCIC paradigms. These biochemical findings highly corroborate extant research at home and abroad on DTT's capacity to improve neuroplasticity (Hao et al. 2025), providing translational validation for clinical implementation. Moreover, the RCIC program demonstrated favorable safety profiles, with significantly lower overall complication rates relative to conventional care despite comparable fall recurrence rates. Superior nursing satisfaction outcomes further reflect advantages in patient‐centered communication, perceived treatment efficacy, and overall care experience, which are of considerable value for healthcare quality assessment and service optimization.

Nevertheless, several methodological limitations warrant acknowledgment. The single‐center design and modest sample size necessitate caution regarding generalizability; thus, future multicenter trials with larger cohorts are recommended for validation. Given that multiple secondary outcomes were analyzed without formal multiplicity adjustment, the risk of Type I error is increased; accordingly, the present findings should serve as hypotheses for future confirmatory studies. Importantly, the short follow‐up window precludes assessment of durability; we recommend future studies incorporating 6‐ or 12‐month follow‐up to evaluate long‐term maintenance of benefits. While short‐term efficacy was established, longitudinal follow‐up is required to evaluate cognitive sustainability and fracture recurrence patterns. Reliance on subjective measures like MoCA could be complemented by objective neurophysiological assessments, such as functional neuroimaging, event‐related potentials (ERPs), in subsequent investigations. It is acknowledged that peripheral serum levels of BDNF, GDNF, S100‐β, and IL‐6 are indirect proxies of central nervous system activity; therefore, these biomarkers should be interpreted with caution and ideally complemented by central measures in future studies.

In conclusion, the RCIC program developed in this study demonstrates significant efficacy in enhancing functional recovery, cognitive performance, and psychological adjustment among elderly patients with lower limb fractures and comorbid cognitive impairment. Its mechanisms likely involve upregulation of neurotrophic factors and suppression of neuroinflammatory cascades. The favorable safety profile and feasibility support its clinical implementation. We advocate for broader adoption in nursing practice and recommend multicenter studies to refine the intervention model, thereby advancing comprehensive and precise care paradigms for geriatric fracture rehabilitation.

## Author Contributions

Q.C. and X.D. contributed equally to the conceptualization and design of the study. Q.C. was primarily responsible for data collection and analysis, while X.D. played a key role in interpreting the results and drafting the manuscript. Y.W., J.W., and M.Z. contributed to data analysis and interpretation, and participated in writing and revising the manuscript. They also provided valuable feedback on the study's methodology and analysis. Yuanyuan Chen and Yanlin Chen contributed to the project's overall management and provided critical feedback on the study's design and implementation. They also contributed to writing and revising the manuscript. All authors played significant roles in the development of the study and approved the final version of the manuscript.

## Funding

The authors have nothing to report.

## Ethics Statement

This study was approved by the Human Ethics Committee of The First Affiliated Hospital of Lishui University. All procedures performed in studies involving human participants were in accordance with the ethical standards of the institutional and/or national research committee and with the 1964 Helsinki declaration and its later amendments or comparable ethical standards.

## Consent

Informed consent was obtained from all individual participants included in the study.

## Conflicts of Interest

The authors declare no conflicts of interest.

## Supporting information




**Supplementary Appendix**: brb371184‐sup‐0001‐AppendixS1.pdf

## Data Availability

The data used and/or analyzed during the current study are available from the corresponding author.
