# Supplementary material for: Rehabilitation–Cognition Integrated Care Program for Elderly With Lower Limb Fractures and Cognitive Impairment: Development and Efficacy
Source: Brain Behav. 2026 Jan 15;16(1):e71184. doi: 10.1002/brb3.71184 (PMC12808919; doi:10.1002/brb3.71184)
Supplement: Supplementary file 1 — Supplementary Appendix: brb371184‐sup‐0001‐AppendixS1.pdf [file BRB3-16-e71184-s001.pdf]

Cognitive-motor Task Manual
(Family Simplified Version)

(For RCIC individualized family practice and family member record-keeping

I. Instructions for Use

This manual is for use by family members and patients during home training during hospitalization and after discharge, covering daily combined training tasks such as orientation, memory, attention, and walking/strength/balance. Safety should be the top priority during training. If any discomfort occurs (dizziness, chest tightness, shortness of breath, wound pain >3/10), stop immediately and contact medical staff.

Ii. Safety and Environmental Preparation

1) Ensure that the walking route is smooth and free of debris, and wear anti-slip shoes. Be sure to check the stability when using a walking aid.

2) during the training need to have a family in the care, prepare a chair to sit down at any time.

3) to prepare a large calendar, pointer clock, direction indicating sticker with nouns CARDS.

4) each measure heart/blood pressure before and after the training (if families have equipment), record the feelings.

Iii. Task Classification and Advancement (Stratified by MoCA)

1) Low-level (MoCA 16-19) : Mainly static balance and simple dual tasks, gradually transitioning to corridor "walk - stop - answer" tasks.

2) Higher level (MoCA 20-25) : Perform complex balancing and route planning tasks in simulated daily life (ADL) scenarios, along with computational/memory challenges.

3) Advanced rules: If the task completion rate is ⩾90% for three consecutive days, the subjective effort is ⩽ moderate (Borg 11-13), and there is no fall or near fall, the difficulty or duration can be increased.

Iv. Daily Recommended Schedule

1) Morning (10-15 minutes) : Orienteering test + memory warm-up (recall 3 words).

2) Morning (15-25 minutes) : Walking + cognitive dual tasks (see Part 6).

3) Afternoon (15-20 minutes) : Strength and joint range of motion training + multi-step instructions.

4) Evening (10 minutes) : Mindful breathing + Recall three events of the day.

V. Cognitive Training (Can be carried out simultaneously with Exercise)

A. Orienteering Training: Ask, "What date is it today?" What time is it now approximately? Which floor am I on? What's the next plan?

B. Attention/Execution Function: Three-step command (lift leg → hold for 3 seconds → lower); Select the category (Fruits/Animals/Daily Necessities).

C. Memory Training: Word Learning and delayed Recall (3 to 5 nouns); Route Recall (Key Points from ward to Nursing Station).

D. Computational Training: Subtract 3 each time starting from 20 (or subtract 2 from 30; adjust the difficulty according to the situation).

Vi. Physical Training (Synchronized or alternating with Cognitive Tasks)

1) Walking enhancement (walker/family member support) : Walk 5 steps, stop and answer orientation questions → Walk another 5 steps; Gradually extend it to 10 to 15 steps.

2) Strength and joint range of motion:

• Early stage (1-2 weeks after surgery) : isometric contraction, simultaneous count 1-5; Each movement consists of 3 sets ×10 repetitions.

• Mid-term (3-6 weeks) : Hip abduction/knee extension with elastic bands, combined with multi-step commands; 2 sets ×8 to 12 repetitions.

• Advanced stage (7-12 weeks) : Stand on one foot on a balance mat (handrail) and recall three words at the same time; Hold for 10 to 20 seconds ×4 to 6 times.

3) Balance and Scenario Simulation: Corridor "Walk - Stop - Answer questions" Walking with a cup and going up and down low steps in the rehabilitation room; Identification and Correction of Incorrect Placement of ADL at Home.

Vii. Brief Mindfulness Intervention (10 Minutes in the evening)

• Sit with your feet touching the ground. Inhale slowly for 4 beats and exhale for 6 beats. Hold this position for 3 to 5 minutes.

• Positive imagery: Imagine walking steadily on a park path, paying attention to synchronizing the rhythm of your steps with your breathing.

Viii. Daily Record Sheet of Family Members

Date	Directional (complete/error)	minutes' walk/nearly fall	Power ROM (group /x)	Memory/Computation (%)	Perceived exertion (Borg)	discomfort

Ix. Weekly Review and Adjustment

Nurses should conduct offline or video follow-ups once a week. If the completion rate is ≥90% and it is safe, the walking time/number of obstacles can be increased, oral prompts shortened, and the number of memorized words increased. If you feel tired or experience a near-fall, reduce the difficulty or switch to a sitting task.

X. Completion of Standards and Connections

After 12 consecutive weeks of training, re-evaluate FMA/BBS/MoCA/FIM, etc. If you need assistance, please contact the responsible nurse or therapist.
